# “It was clear to me that nothing else would make me happy” – a qualitative, type-building interview study on meaningfulness and motivation in medical studies at two medical faculties

**DOI:** 10.3205/zma001835

**Published:** 2026-03-23

**Authors:** Felix Albrecht, Claudia Kiessling, Gina Atzeni, Pascal O. Berberat, Paula Matcau, Gabriele Lutz

**Affiliations:** 1 Witten/Herdecke University, Faculty of Health, Witten, Germany; 2 Witten/Herdecke University, Faculty of Health, Education of Personal and Interpersonal Competencies in Healthcare, Witten, Germany; 3 Ludwig-Maximilians-Universität Munich, Institute of Sociology, Munich, Germany; 4 Technical University of Munich, TUM School of Medicine and Health, Department of Clinical Medicine, TUM Medical Education Center, Munich, Germany

**Keywords:** experience of meaningfulness, crises of meaning, search for meaning, types of meaning, motivation, medical education, medical students

## Abstract

**Objective:**

The aim of this study was to qualitatively describe the relationships between the experience of meaningfulness, crises of meaning and the motivation of medical students.

**Methods:**

Semi-structured interviews using an interview guide with 20 students in the clinical study phase were analyzed using type-building qualitative content analysis according to Kuckartz.

**Results:**

Sources of professional meaning were identified as belonging and teamwork, performance and striving for development, autonomy, coherence and social relevance. Transition phases, pressure to perform, negative practical experiences and professional or system-related challenges were cited as inhibiting factors for a good experience of meaningfulness.

The students reported that experiences of meaningfulness and crises of meaning had an impact on their motivation. Four specific student-types were elaborated, regarding the degree of motivation for their studies and later career entry, in professional meaningfulness and crises: *Students with a calling* had the strongest motivation and the highest experience of meaningfulness with hardly any crises of meaning. *Students who are seekers* had a high experience of meaningfulness, but their motivation was low due to crises of meaning, in contrast to *students who are doubters*, who also had a low experience of meaningfulness. *Students who are work-to-live persons*, despite low levels of experience of meaningfulness or crises of meaning, showed motivation for their studies and later careers.

**Conclusion:**

Students’ experiences of meaningfulness and crises of meaning were shown to be related to their motivation to study and later career entry. The proposed typology revealed individual accentuations of the students on how they can cope with experiences of meaningfulness and motivation. Further research is required to confirm the relationships found and to develop targeted interventions based on them.

## Zusammenfassung

**Zielsetzung:**

Ziel der vorliegenden Untersuchung war es, Zusammenhänge zwischen Sinnerleben, Sinnkrisen und der Motivation von Medizinstudierenden qualitativ zu beschreiben.

**Methoden:**

Leitfadengestützte Interviews mit 20 Studierenden im klinischen Studienabschnitt wurden mittels typenbildender qualitativer Inhaltsanalyse nach Kuckartz ausgewertet.

**Ergebnisse:**

Als berufliche Sinnquellen konnten Zugehörigkeit und Teamarbeit, Leistung und Entwicklungsstreben, Autonomie, Kohärenz und gesellschaftliche Relevanz identifiziert werden. Als hemmende Faktoren des Sinnerlebens wurden Transitionsphasen, Leistungsdruck, negative Lernerfahrungen und professions- oder systembezogene Herausforderungen genannt.

Die Studierenden berichteten, dass sich Sinnerleben und Sinnkrisen auf ihre Motivation auswirkte. Hier konnten vier spezifische Typen identifiziert werden, die sich in der Ausprägung der Motivation für das Studium und den späteren Berufseinstieg, in beruflichem Sinnerleben und -krisen, akzentuieren. *Die Berufenen* hatten die stärkste Motivation und ein hohes Sinnerleben bei kaum vorhandenen Sinnkrisen. Bei *den Suchenden* war das Sinnerleben hoch, jedoch durch Sinnkrisen die Motivation gering, im Gegensatz zu *den Zweifelnden*, bei denen auch das Sinnerleben geringer war. *Jobber* zeigten trotz geringem Sinnerleben oder -krisen Motivation für das Studium und den späteren Beruf.

**Schlussfolgerung:**

Es konnte gezeigt werden, dass die Motivation für das Studium und den späteren Berufseinstieg mit dem Sinnerleben und -krisen der Studierenden verbunden war. Die vorgeschlagene Typenbildung zeigt individuelle Akzentuierungen der Studierenden. Es bedarf weiterer Forschung, um die gefundenen Zusammenhänge zu bestätigen und daraus gezielte Interventionen abzuleiten.

## 1. Introduction

Seeking and finding meaning is a central element of human existence [1] and can influence people's motivation [2]. In scientific literature, meaning is defined in different ways. Based on existing definitions of meaning, Martela and colleagues proposed the following three essential commonalities for a definition: *Significance, purpose* and *coherence* [3]. The experience of meaningfulness can consist firstly of the subjective *significance* that a person attaches to a thing or event [4], secondly of an overarching *purpose*, so that life content is geared towards achieving goals [5] and thirdly of the feeling of a *consistent connection* with oneself and the environment, also known as coherence [2], [6], [7], [8]. Sources of meaning are individual, diverse and dynamic [5]. For example, many people find social interaction, spirituality or nature experiences meaningful [5], [9]. Meaningfulness is an everyday, tangible [10], [11] but overall rather unconscious experience. A conscious exploration of questions regarding meaning usually requires an external trigger, such as critical life events or transitional situations into new life phases, so-called transition phases [5], [12]. In some cases, these challenges are perceived as crises of meaning, which are defined as a lack of meaning combined with a longing for meaning [5]. A crisis of meaning can occur independently of an experience of meaningfulness, meaning that the lack of meaning does not necessarily lead to a crisis of meaning [13] but can also be an expression of indifference.

Based on this, Schnell and colleagues developed a system with four types [5], which is characterized by the juxtaposition of the experience of meaningfulness and crises of meaning. The type “meaningful” describes high meaningfulness and low crisis of meaning, while the experience of high crisis of meaning and low meaningfulness is described as the type “crisis of meaning”. The simultaneous experience of meaningfulness and crisis of meaning was defined as the type “conflicting” [5], [13]. The group that reported a low crisis of meaning and low meaningfulness was described as “existentially indifferent”. Various characteristics could be assigned to this type, such as generally low competence values, little commitment or little interest in self-knowledge [5].

While the importance of meaningfulness in private life has been known for some time [14], meaning in professional life has only become the focus of research in recent years [15], [16]. Among other things, the following sources of professional meaning have been identified: Alignment of personal values with organizational values, the opportunity for autonomous responsibility and decision-making, contributing to society and personal development in a professional context [5], [17], [18], [19]. A high sense of professional meaningfulness correlated with greater efficiency, higher work commitment, and increased motivation [5], [15], [20], [21], [22].

Research on the experience of meaningfulness in medical studies has been scarce. Initial findings in the context of professional identity formation (PIF) indicate that the experience of meaningfulness in medical training can play a role in students’ well-being and influence how they cope with conflicts of values [23], [24]. It is also known that motivation is particularly high at the beginning of studies [25], [26]. Motivation has been identified as a key factor for learning success [27] and academic success [26]. By definition, motivation can be understood as a phenomenon that is influenced by self-perception and perception of the environment. It leads individuals to make the decision to start, maintain and learn from an activity [27]. There are contradictory findings about the further development of motivation during the course of study [28], [29]. Whether and how study motivation and the experience of meaningfulness during medical education are related has not been the subject of research yet. In a quantitative survey that preceded this study, insights into medical students’ experiences of meaning were explored for the first time. The survey found that most of the respondents had already dealt with questions of meaning in relation to their studies and in contact with patients. The experience of meaningfulness was rated lower by students at the end of their studies than by students in the middle of their studies, for which criticism of study and framework conditions was named as the main cause [30]. While the topic of meaningful life as a health-relevant factor was formulated as a learning objective in the new National Competence-Based Catalogue of Learning Objectives in Medicine 2.0 [https://nklm.de/zend/menu], it is not known to what extent it is already addressed in existing medical curricula.

The aim of this study was to investigate the deeper understanding of medical students’ meaningfulness through qualitative study. The focus was on the role of experiences of meaningfulness and crises of meaning, and the influence of experiences during their studies on their experience of meaningfulness and motivation for their studies and later careers.

## 2. Methods

### 2.1. Study design and setting

We conducted an interview study at two German medical faculties with medical students at the beginning and end of the clinical study phase. This approach was chosen in order to obtain perspectives that were as heterogeneous as possible and to take into account the fact that medical programs can differ in terms of content, study conditions and the hidden curriculum [31]. The private University of Witten/Herdecke (UW/H) offers a model curriculum for currently around 84 students per semester with early patient contact in the preclinical phase. The Technical University of Munich (TUM) offers a standard curriculum with around 175 students per semester.

### 2.2. Instruments used

The research team developed an interview guide for data collection in the winter semester 2021/22. In several sessions, FA (F. Albrecht), CK (C. Kiessling) and GL (G. Lutz) developed questions to explore the students’ perspectives on the research questions. The guide was then discussed, agreed and subsequently piloted as part of an external audit with GA (G. Atzeni), PM (P. Matcau) and PB (P. Berberat). For example, personal and professional backgrounds, such as teaching assignments, were reflected upon to ensure that the questions were formulated openly and as non-judgmentally as possible. The interview guide contained 14 questions (see attachment 1 ) and addressed, on the one hand, dealing with one's own questions of meaning and one’s own experience of meaningfulness and, on the other hand, dealing with patients’ questions of meaning. This article focuses on dealing with one’s own experience of meaningfulness and crises of meaning. In addition, the following personal data was collected: place of study (TUM, UW/H), semester, gender, age, previous engagement with questions of meaning. 

### 2.3. Recruitment and data collection

Recruitment for the interview study took place as part of the aforementioned quantitative survey [30]. We contacted all medical students in the 5^th^, 6^th^, 10^th^ and 11^th^ semester at UW/H and TUM three times by e-mail through the respective deans of student affairs together with information about the study at two-week intervals at the beginning of the summer semester 2022, independently of courses. A total of 940 students were able to participate in the survey as the target population (700 TUM/240 UW/H). First, an explorative, quantitative cross-sectional survey with a total of 9 questions was conducted to gain initial knowledge of the research field [30]. Of the 111 participants, 44 students expressed interest in an additional interview. Of these, 20 students were selected in such a way that the study population was as heterogeneous as possible in terms of location, stage of study, gender and engagement with meaningful experience. The interviews were conducted either by telephone or via ZOOM and lasted between 46 and 103 minutes (75 minutes on average). 

### 2.4. Data analysis 

The interviews were recorded and then transcribed verbatim, while all personal data was anonymized. We analyzed the interviews using qualitative content analysis according to Kuckartz and Rädiker [32]. The interview questions provided an initial basic framework of structuring, deductive, superordinate categories. In a subsequent step, FA, CK and GL read and inductively coded the first four interviews independently of each other.

First categories and initial impressions about relationships between the categories were continuously recorded in memos and considered in the analysis. We created additional case summaries to work out central aspects. The initial coding created a first level of abstraction. 

After defining the first main categories and subcategories, we created a preliminary concept map, resulting in an initial category system. The categories and concept map were then discussed with GA, PM and PB with regard to internal homogeneity and external heterogeneity in the sense of an external audit. The other interviews were then coded using FA’s category system. In a subsequent run-through, the content of some existing categories was expanded, details were added, and definitions were provided. Newly emerging topics were included in new categories.

Once all the interviews had been coded, the adapted concept map was first discussed by the research team. The elaborations and preliminary definitions were discussed repeatedly based on the data material until a consensus was reached. This final category system was then reapplied to all interviews. Based on the final category system, we started to build a typology [32] and created a concept map of the central categories to visualize the relationships between the categories and to identify the essential characteristics for type building. This served as the basis for developing the relationships between motivation, meaningful life and meaning crises. First, similarities and differences in the experience of meaningfulness and in the perception of crisis of meaning were examined. Based on the case summaries, the interviews were sorted and grouped according to several dichotomous characteristics, such as degree of meaningfulness experience (low/high), crisis of meaning (barely present/expressed), and motivation (low/high). Based on these groupings, a natural type building was carried out in which similarities and differences in the characteristics were systematically analyzed. Finally, the results of the evaluation were presented descriptively and visually. 

In each phase, reflection took place on personal, interpersonal, methodological and contextual factors that could influence the analysis of the data [33]. For example, the team exchanged their own sources and crises of meaning, different preferences for the design of medical programs and affiliation to one of the two locations, different disciplinary understandings of meaning as well as biographical and intergenerational perspectives. The standards for reporting qualitative research (SRQR) were used in the preparation of the manuscript [34].

### 2.5. Ethics approval

No ethical concerns were identified by the UW/H Ethics Committee (application no. S-51/2022). The TUM Ethics Committee concurred with the vote (2022-215-S).

## 3. Results

### 3.1. Description of the study population

A total of 20 students participated in the study. Personal characteristics are described in table 1 (Tab. 1).

### 3.2. Results of the data analysis 

The results report is structured according to the central categories and the built typology. Categories and subcategories are printed in italics in headings or in the body text; quotations are included in the text in quotation marks for illustrative purposes; further quotations are added in table 2 (Tab. 2) and table 3 (Tab. 3). 

#### 3.2.1. How do students experience meaning during their studies? – Sources of meaning

When asked about an experience of meaningfulness related to their studies, five overarching sources of meaning were reported that could contribute to a meaningful experience: On the one hand, it was meaningful when students felt *part of a team* “that I can work well with” (#15) during their medical education and first practical activities and were able to make a contribution to the team “so that it works in the end” (#20). Among other things, *striving for performance and development *was frequently formulated. Particularly in the preclinical phase, the focus was on acquiring knowledge and gaining a deeper understanding of the human body. Even if not for everyone, the successful application of medical skills, such as inserting venous catheters, was added as a source of meaning as students progressed. For example, “the activity itself [...] and also seeing how you get better at this activity” (#14) was described as meaningful. Also important was an increase in *autonomy* in the sense of gradually taking on responsibility and involvement in practice by taking on one’s own tasks: “[...] like being on a safety line” (#2). Flat hierarchies and the opportunity to participate in the design of the course also contributed to an experience of meaningfulness. At the same time, *coherence* between one’s own aspirations and values and those that could be experienced in patient care was important. The caring aspect of the relationship led most of them to a meaningful sense of doing something that had *social relevance*: “a contribution to the lives of others [...] where I can say that I have been helpful or useful” (#14).

#### 3.2.2. How is meaning experienced? 

Overall, students showed a mixed picture regarding the extent to which their studies contributed to experiencing meaning. The overall impression was that half of them found their studies meaningful. A high experience of meaningfulness was expressed by the students through a feeling of satisfaction and arriving in the moment. Most attributed positive emotional experiences such as joy and fun, gratitude, self-affirmation and self-efficacy to a experience of meaningfulness, as well as long-term positive effects on motivation.

#### 3.2.3. What are the factors that inhibit the experience of meaningfulness?

We categorized factors that had a negative impact on the experience of meaningfulness as inhibiting factors on the experience of meaningfulness. In most cases, they provoked a conscious confrontation with questions of meaning or even could trigger a crisis of meaning, which was defined by a crisis-like experience and a conscious confrontation with questions of meaning. Four categories of inhibiting factors could be identified: On the one hand,* transition phases*, such as the decision to go to university, could lead to a negative or even crisis-like experience, as these were often accompanied by a “lack of orientation” (#3) and “fear of the future” (#3). Furthermore, transitions into new phases of education were characterized by specific uncertainties. For example, learning strategies no longer worked after the first part of the medical state examination (“Physikum”) due to the increased amount of material. 

On the other hand, the *pressure to perform and the type of learning* led to crises of meaning. The “incredibly high work and study load” (#6), “this mindless memorization” (#15), sometimes without any recognizable practical relevance, and the pressure to pass exams had a negative impact. This was exacerbated by the simultaneous social pressure to meet the demands of their environment.

Negative *practical learning experiences*, such as primary use for blood sampling or supporting activities in the operating theater, were perceived as exploitation. Often, bedside teaching had to be actively fought for. In some cases, there was no room for students’ doubts and criticism, or they were not taken seriously.

The *confrontation with professional and system-related challenges* also led to crisis-like experiences. Throughout the course of medical training, there was rarely room for reflection on the understanding of roles or an honest approach to dealing with high workloads, which made students feel left alone. The experienced staff shortages, frequent frustrations, complete exhaustion and demotivation among practicing doctors were accompanied by fears for their own survival and health in the German healthcare system. The desire for positive role models was central here. In addition, their own ideals of patient-centered care were repeatedly burdened by a system based on DRGs (Diagnosis Related Groups) that they experienced as dehumanizing. The “complete economization of the system” (#6) creates ethical dilemmas, as patient care is often “really just a symptom treatment” (#12).

#### 3.2.4. What effect do crises of meaning have?

Students indicated that inhibiting factors in their experience of meaningfulness led to a crisis-like experience. In two cases, lasting doubts about the career choice were stated. For many, the experience of crises of meaning was directly demotivating, frustrating and characterized by excessive demands and helplessness.

#### 3.2.5. How was the motivation for medical studies and later career described?

While the initial motivation of some students suffered because of their first practical experience, it increased again for these students as they developed a sense of belonging and identification with an area of specialization. Some students appeared to be hardly influenced and were predominantly highly motivated. High motivation was expressed by an optimistic view of the future, enjoyment of work and existing coping strategies for challenging situations in the healthcare sector. A rather pessimistic view of the future career path and the description of numerous doubts that would go hand in hand with professional practice, without finding solutions, characterized a decreasing or lower motivation. 

#### 3.2.6. Typology

**Figure 1:**
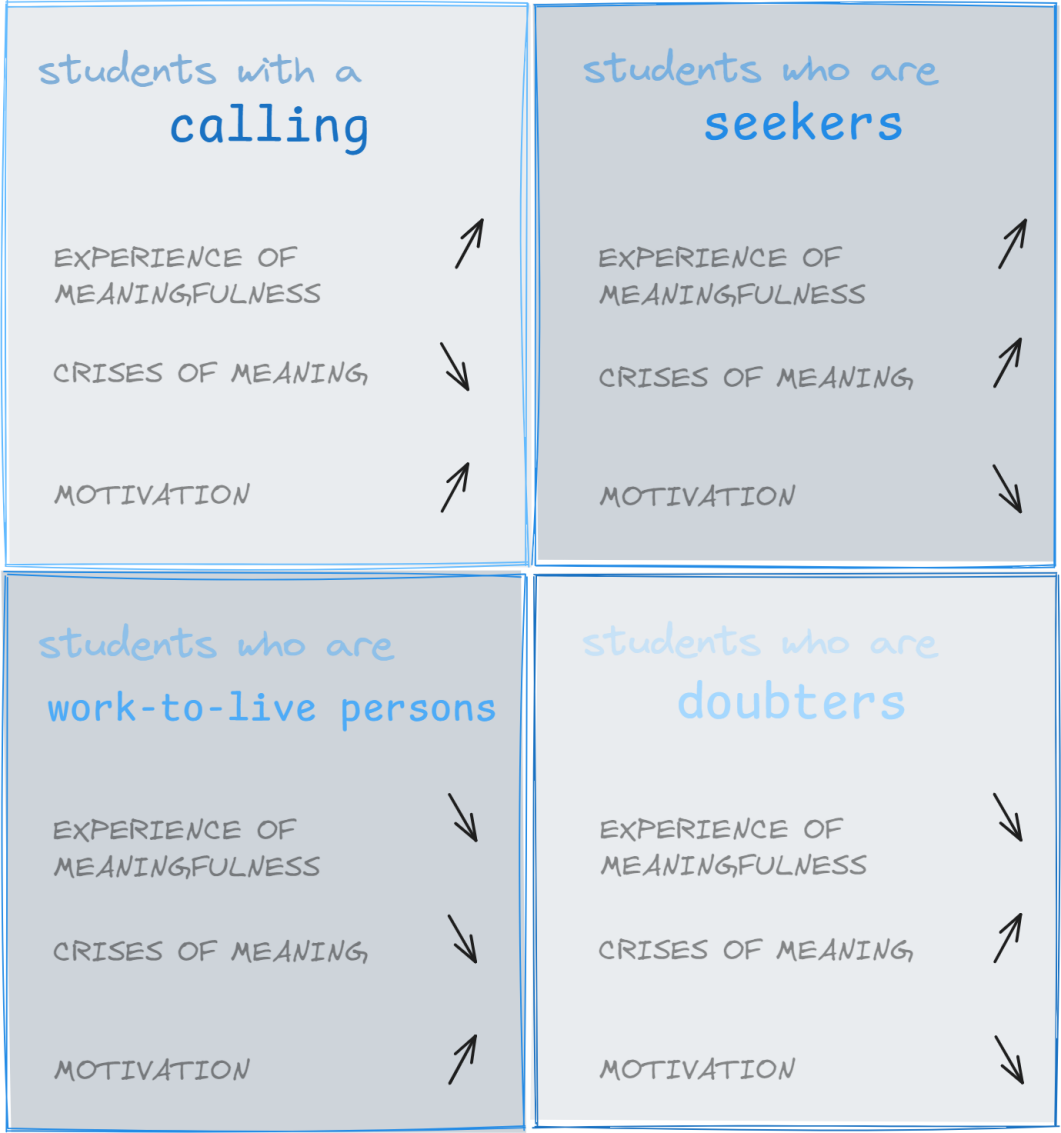
Illustrative representation of the type formation with the central, study/occupation-related categories

By grouping the interviews according to similarities in the level of meaningfulness, a connection between motivation, experience of meaningfulness and crises of meaning could be identified. Depending on the extent of the experience of meaningfulness and crisis of meaning, four types were built, which also differed in their motivation: *students with a calling, who are work-to-live persons, who are seekers and who are doubters.* Figure 1 (Fig. 1) shows a visualized representation. Table 4 (Tab. 4) contains exemplary quotations for the individual types.

*Students with a calling* seemed to have a strong experience of meaningfulness and did not allow themselves to be led astray by factors that inhibited their experience of meaningfulness, so they did not experience any crises of meaning. This apparently resulted in long-term and high motivation. The focus of *the students who are work-to-live persons* was on performing well. Inhibiting factors were sometimes perceived, but did not lead to a crisis-like experience. Rather, the job was subordinated to the personal construct of meaning in the sense of a balanced work-life balance. Questions of meaning played a subordinate role overall. The start of the career was viewed positively. While *students who are seekers* had a high experience of meaningfulness for parts of their studies, there were also crises of meaning. This apparent inner conflict seemed to preoccupy the interviewees of this type so much that their motivation to continue suffered as a result. Compared to the other types, *students who are seekers* were younger and, like the *students who are work-to-live persons*, had only dealt with questions of meaning on an irregular basis. *Students who are doubters*, on the other hand, only experienced an experience of meaningfulness to a limited extent, as it was overshadowed by crises of meaning. Motivation to study was less pronounced. In the location comparison, there tended to be more *students who are work-to-live persons at TUM (5 TUM, 3 UW/H), while the two students who are doubters* were at UW/H. The assignment of students to a type of meaning can change due to changes in their experience of meaningfulness and should be understood as a dynamic process.

## 4. Discussion

The aim of the study was to investigate for the first time the perspectives and relationship between motivation, experience of meaningfulness and crises of meaning in the context of medical studies using qualitative interviews. 

Five overarching areas were identified as sources of meaning: Belonging & teamwork, performance & striving for development, autonomy, coherence, and social relevance, which can be found in similar form in the literature [5], [17], [18], [19]. We were able to identify specific factors that inhibit the experience of meaningfulness, such as transition phases, pressure to perform, negative practical learning experiences and professional or system-related challenges. These could provide an explanation as to why so many people working in the healthcare system are frustrated, want to reduce their working hours or even leave [35], [36]. According to the students’ reports, motivation was subject to changes in both positive and negative directions. 

Overall, we were able to identify a relationship between experience of meaningfulness, crises of meaning and motivation, which has already been described both in the general [20], [21], [22], [37] as well as in the professional context [15], [36], [38]. An experience of meaningfulness seemed to have a positive effect on the motivation to study, while inhibiting factors had a negative effect. Contrary to previous assumptions [5], our analyses suggest that crises of meaning may have a stronger influence on immediate motivation. This became evident, as inhibiting factors could directly affect motivation and career choice in cases of low meaningfulness. According to our typology, this applies to *students who are work-to-live persons* and* students who doubt*. A positive experience of meaningfulness seemed to have a more long-term effect and should be discussed as a protective factor. According to the students’ reports, meaningful moments sometimes contributed to motivation for months and helped to overcome crises of meaning and phases of doubt. As our results are temporary impressions, it would be important to further investigate the medium-term and long-term consequences of a lack of meaningfulness or crises of meaning over the long term. 

With regard to the type-building we developed, we were able to find parallels and differences to the types Schnell and colleagues have developed [5], [39]. This could be since our focus was on a specific student context, which is why we propose different types for this context. Also new and different in our context is the integration of motivation into the type-building, which offers added value in terms of content, especially for *students who are seekers* and *students who are work-to-live persons*. While the clear association described in the literature between high/low experience of meaningfulness and correspondingly high/low motivation [15] was found for *students with a calling* or *students who are doubters*, this is different for the other two types:

*Students who are seekers* seemed to experience an inner conflict due to the simultaneous occurrence of an experience of meaningfulness and a crisis of meaning. The students described this as a decline in motivation for their studies. Schnell et al. refer to the simultaneous occurrence of experience of meaningfulness and crises of meaning as the “contradictory type” [5] but do not link this to motivational factors and describe the presence of two contradictory experiences as “implausible” ([35], p.7). In a further study, Schnell and colleagues were able to show that the experience of meaningfulness and crises of meaning are quite independent of each other [13]. Our work underlines this and allows the assumption that a simultaneous occurrence is not contradictory, but rather a search for congruence and resolution of the presumably prevailing conflict. *Students who are seekers* described how the search resulted in resignation with lasting doubts when they had lost sight of the meaningful potential of their career aspirations due to a variety of inhibiting factors. Because the experience of meaningfulness develops dynamically over the course of life, assigning students to a particular meaning type is generally only possible at specific points in time [5].

The extent to which the type *of students who are work-to-live persons* we have defined corresponds to the type of existential indifference according to Schnell and colleagues remains to be discussed. Schnell et al. attribute avoidance behavior to the existential indifference type in order to avoid anxiety and unpleasant situations [5]. Similar distancing mechanisms have already been observed in the decline in empathy and the development of cynicism and burnout in trainee doctors [40] [41], [42]. *The workers’* sporadic engagement with questions of meaning coupled with high motivation raises the question of whether they may deliberately avoid dealing with experience of meaningfulness and crises of meaning, as this could lead to lasting doubts and possibly to dropping out of their studies. Evidence for this assumption was already found in a previous study [30]. The avoidance of questions of meaning as protection against unpleasant confrontations, for example with one’s own role in the healthcare system, should be considered in further studies. Among *students who are work-to-live persons*, meaning seemed to be sought primarily in the private sphere, which could explain why there seems to be a shift in priorities towards more private life in constructs such as work-life balance [43]. As the students in this group were highly motivated at the time of their studies, there seems to be no cause for concern at present. However, it remains questionable whether the supposed avoidance of issues related to the meaning of medical education can maintain the motivation of future medical doctors in the long term. Further research will be necessary to find out whether motivation remains stable at a high level in the further career of these students.

In our cohort, the younger students were *seeking* meaning in their medical studies, sometimes accompanied by an inner conflict, regardless of how far they had progressed in their course of studies. The older students had apparently either found their *calling, doubted* their career choice or had subordinated the profession to their individual construct of meaning (cf. *students who are work-to-live persons*). This indicates that the experience of professional meaning is classified as part of the overall construct “meaning in one’s own life” depending on age. Especially at the beginning of their studies and younger students in general could be supported in this way by learning activities aimed at identifying individual sources of meaning. In the long term,* students who are work-to-live persons* could also benefit if they could experience more meaningfulness. In the case of *students who are seekers* and *students who are doubters*, support in dealing with inhibiting factors would appear to be particularly useful to actively help them overcome crises of meaning. The extent to which our proposed type-building offers helpful approaches for designing learning activities should be addressed in further work. It is already clear from the available data that there will not be a one-fits-all solution for all students. In our view, the available evidence on students’ experiences of meaningfulness is promising and should be pursued in further work.

### 4.1. Limitations

Due to voluntary participation in the study, there could be a selection bias, as it cannot be ruled out that a disproportionate number of students who participated consider the experience of meaningfulness and crises to be meaningful. Although the self-assessment of how often the topic is present was considered during the selection process, this subjective assessment is ultimately difficult to objectify. Furthermore, the sample size is relatively small, meaning that generalization to other student cohorts is limited, despite the inclusion of two locations with a model curriculum and a standard curriculum. Despite the age difference between the location cohorts, no differences in meaningfulness could be identified. The collection of cross-sectional data only allows limited conclusions about progression in the clinical phase. As there are only a few findings to date on questions of meaning among medical students, further investigations at other locations and as part of longitudinal studies would be desirable in order to validate our results and derive targeted interventions.

## 5. Conclusion

Our study suggests that experiencing positive meaning and consciously exploring sources of meaning in professional life can have a lasting motivational impact on students. According to the students’ statements, inhibiting factors of the experience of meaningfulness had a direct negative impact on their motivation to study and their expectations regarding the start of their careers. The present results suggest that addressing the experience of meaningfulness and strengthening sources of meaning in medical education and later clinical work could serve as a protective factor in crises of meaning and promote the motivation of future doctors in the long term. It makes sense to use the inhibiting factors of the younger generation to systematically explore the critical aspects of medical education and the healthcare system and to improve existing structures. Type-building is a first step towards identifying students’ needs in questions of meaning and in the process of identity formation to address these as they progress. 

## Acknowledgements

We would like to thank Mandy and Michael Williams for their invaluable help in translating the article. We would also like to thank all the students who participated in the extensive study and shared their thoughts and feelings on meaningfulness. Our thanks also go to the deans of studies who sent out recruitment emails.

## Authors’ ORCIDs

- Felix Albrecht: [0000-0001-9927-7090]
- Claudia Kiessling: [0000-0003-4104-4854]
- Gina Atzeni: [0009-0002-9227-3980]
- Pascal Berberat: [0000-0001-5022-5265]
- Paula Matcau: [0009-0007-4119-6328]
- Gabriele Lutz: [0000-0001-5044-8485]

## Competing interests

The authors declare that they have no competing interests. 

## Supplementary Material

Interview guide

## Figures and Tables

**Table 1:**
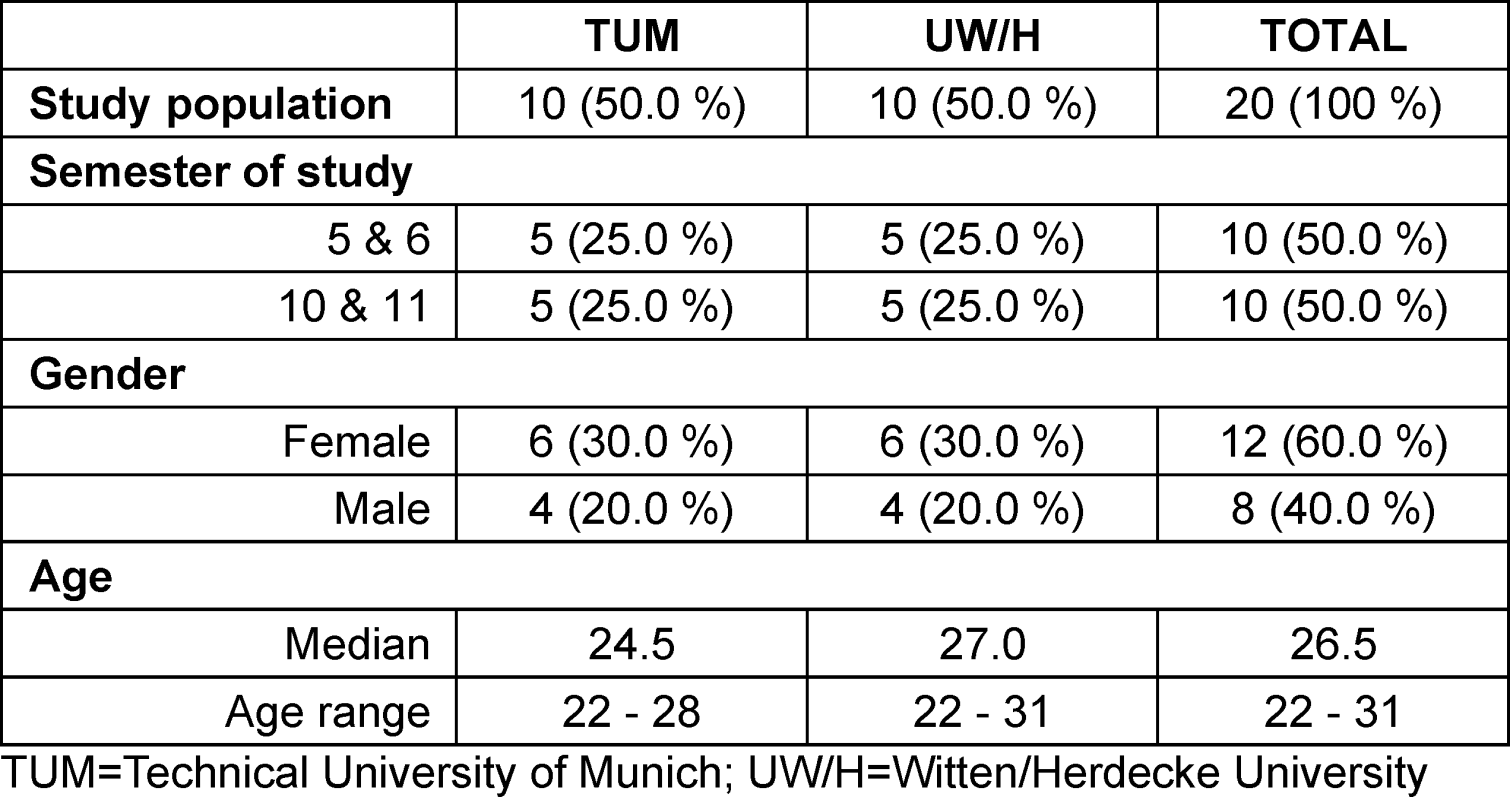
Detailed composition of the study population based on socio-demographic data

**Table 2:**
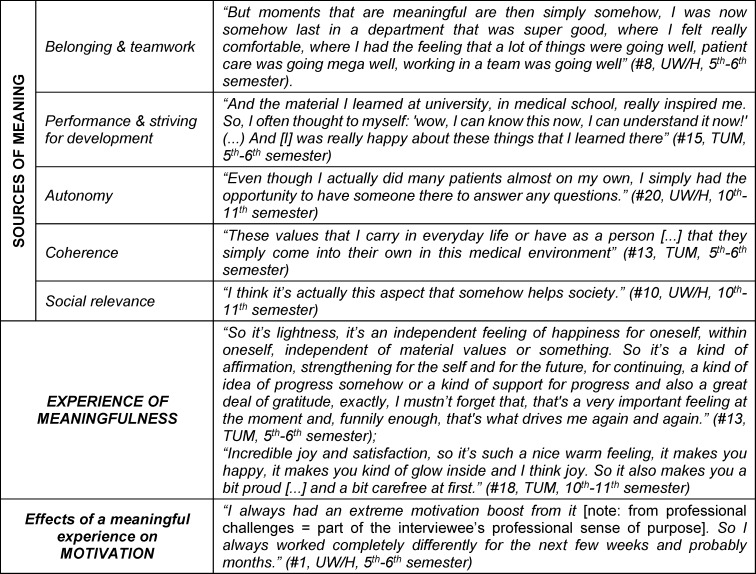
Sources of meaning, experience of meaningfulness and their impact on student motivation

**Table 3:**
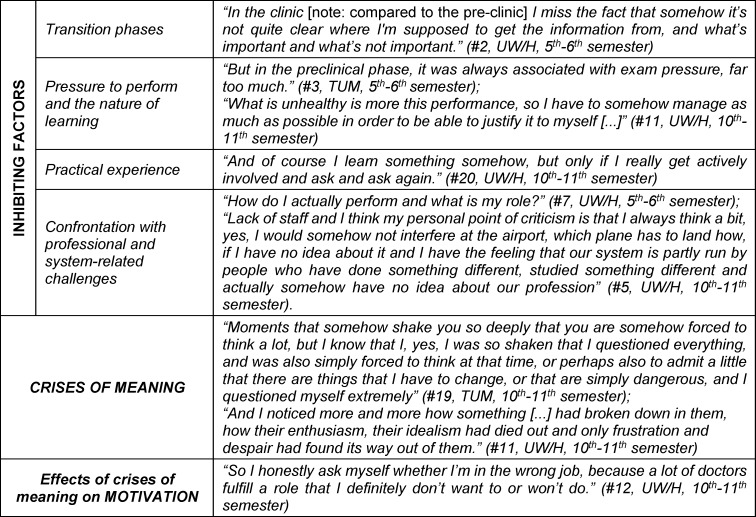
Inhibiting factors in the experience of meaningfulness, crises of meaning and their impact on student motivation

**Table 4:**
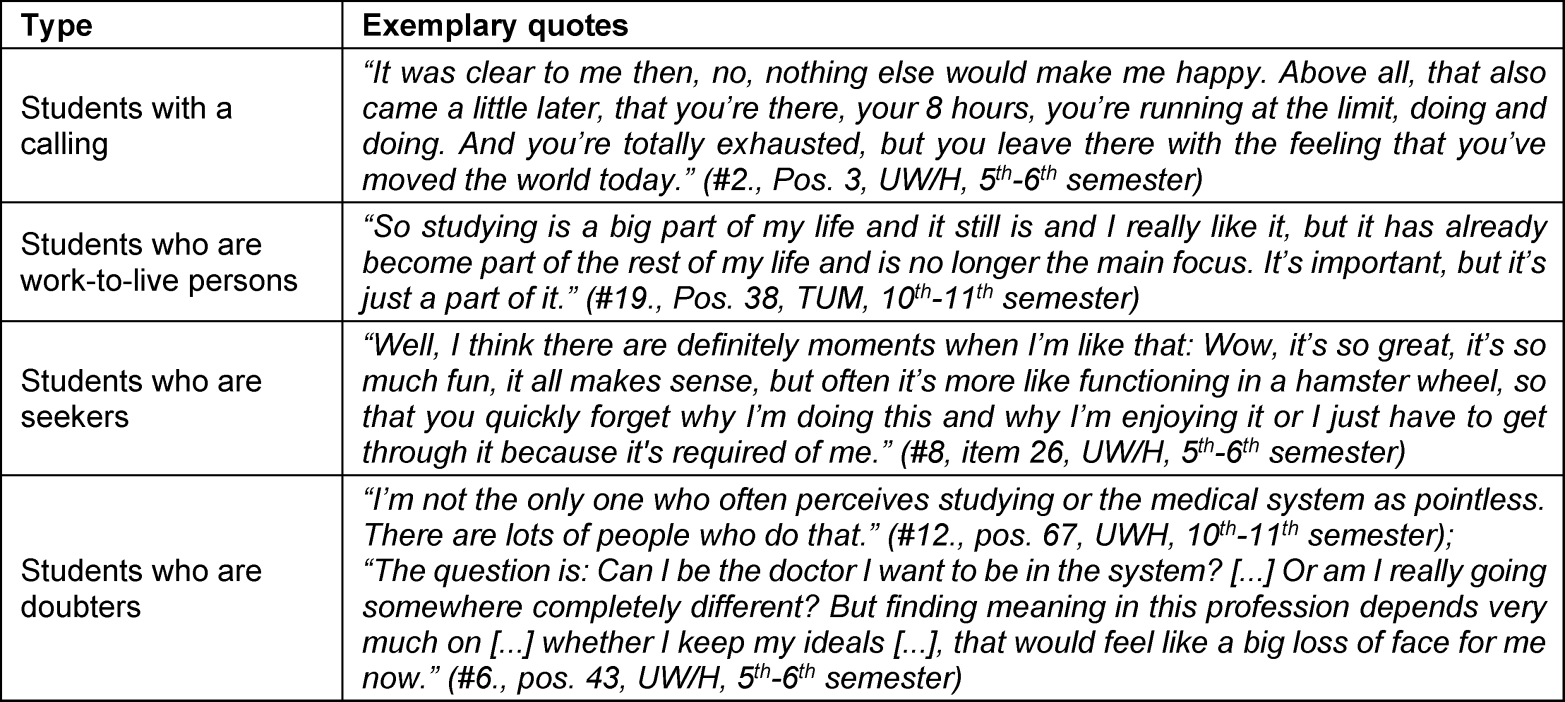
Exemplary quotations of the respective types
