# Supplementary material for: “It was clear to me that nothing else would make me happy” – a qualitative, type-building interview study on meaningfulness and motivation in medical studies at two medical faculties
Source: GMS J Med Educ. 2026 Mar 23;43(3):Doc41. doi: 10.3205/zma001835 (PMC13054799; doi:10.3205/zma001835)
Supplement: Interview guide [file JME-43-41-s-001.pdf]

Attachment 1: Interview guide

GUIDING QUESTION/ NARRATIVE PROMPT<br>Stimulus	COGNITIVE INTEREST<br>Checklist	CONCRETE (follow-up) QUESTIONS<br>Questions in the back of your mind
Dear (NAME), thank you for your interest in this interview.<br><br>*Do you agree to the recording of the following conversation?*<br><br>*Together, we will address some questions about meaning in life and your experience of meaningfulness. In other words, what is important to you personally, how it felt for you, how you dealt with it, and what role your studies played in this. We will talk for about 1 hour, or a little longer.*<br><br>*I would be happy to offer you the first name!*<br><br>*Briefly about myself and my background:*<br>*My name is Felix and I'm currently studying medicine in my 7th semester.*<br>*I am conducting this study at the Chair of Internal Work and Personality Development at the University of Witten/Herdecke in cooperation with the Technical University of Munich, and would like to make a contribution to students and future physicians by researching the importance of meaningful experience.*<br><br>*I would like to let you know in advance that there will be no categories such as right and wrong in the study, especially when it comes to terms such as meaning in life, meaningful life and meaningfulness, which may seem unwieldy at first. What counts for us is your individual view and your experiences of the various aspects so that we can better categorize the process during the course of the study.*<br><br>*It is important to us that we do not want you to justify your views in the planned interview with our questions. With this study, we want to get to know your opinions, thoughts and attitudes without judging them. Many of the motives for meaning may seem very banal to you. But what counts is that it has meaning for you.*<br><br>*If there are any questions that are too intimate or too close to you, please let me know during the course and we can skip them.*	**Thanks**<br><br>**Consent recording**<br><br>**Outlook: in terms of time / content**<br><br>**Offering the first name**<br><br>**Introduction: Chair IAP and myself.**<br><br>**Value of answers, everything is allowed!**<br><br>**TRIGGER WARNING**	

Attachment 1 to Albrecht F, Kiessling C, Atzeni G, Berberat PO, Matcau P, Lutz G. *"It was clear to me that nothing else would make me happy" – a qualitative, type-building interview study on meaningfulness and motivation in medical studies at two medical faculties.* GMS J Med Educ. 2026;43(2):Doc41. DOI: 10.3205/zma001835

A little hint: I may write down little notes during the process, so you don't have to wonder!<br><br>*If you want us to take a short break, just let us know.*<br><br>*Do you have any questions before we start?*		
I would like to start with a question that you have probably heard many times: *"Why did you decide to become a doctor?"*	**Introduction to the topic**	**Why exactly this reason?**<br>**What does it mean to you?**<br>**Motivation? Meaningful? Helpful motive?**
What is meaningful for you?	**How do the participants define meaningfulness for themselves?**	**When does something feel meaningful and meaningful to you?**<br><br>**In your opinion, what is a meaningful life for you personally?**<br><br>**If you imagine that today is your last day, what would you have to have experienced in retrospect to be able to say that you have led a meaningful life?**<br><br>**What would you like to be remembered for when you pass away?**<br><br>**How would you describe your purpose in life?**
*To what extent do you find your **medical studies** meaningful?*<br><br>*To what extent do you find your **day at the clinic** meaningful?*<br><br>*Which elements do you find meaningful?*<br><br>• *When you look back: Has your attitude changed over the course of your studies?*<br>• *Do you think this will change in the course of your studies?*	**Status quo: Does theory make sense?**<br><br>**Status quo: Does everyday hospital life make sense?**<br><br>**Question aims at the concept of meaning! Dimensions of meaning**<br><br>**Looking back?**<br><br>**Outlook?**	**(Activity, dealing with people, friendships, colleagues?)**<br><br>**To what extent do you experience differences?**

Can you remember situations in your life when you had a great sense of purpose?	**Meaningful experience: What does this mean for the participants, approximated via examplee**	**So a situation where you had the feeling, this all makes sense, I'm exactly where I want to be!**<br><br>➔ **In the private sector?**<br>➔ **During your studies?**<br>➔ **In practical experience?**
How did you feel in such situations?	**A sense of purpose?**<br><br>➔ **Effects on the participant?**	**What does that mean to you?**<br>**What did this trigger in the situation described above?**<br><br>**Motivation?**
What role have questions of meaning played in your life so far?<br><br>*Have you noticed a change in the question of meaning in your life so far?*<br><br>*Can you name a trigger?*	**A look into the past:**<br><br>**Change in the course of time**<br><br>**Reasons for a change**	**What role did questions of meaning play before / at the beginning / after your studies?**<br><br>**Was there a trigger that prompted you to deal with the topic? (Family, friends, difficult life situations?)**
Have you experienced something in your life that could be described as ***a crisis of meaning?***<br><br>• *What helped you with this?*<br>• *Would you say it had an impact (in the moment or long term?) on your life, if so, what was it?*<br>• *Are there central thoughts that give you strength to carry on in certain situations?*	**Crises of meaning**<br><br>**Coping strategies.**<br>**Effects, consequences for the future**<br><br>**Patterns**	**Offer a definition of a crisis of meaning?**<br>**-> "Emptiness of meaning with a simultaneous longing for meaning" (Schnell 2004, 2009)**
Do you experience the confrontation with questions of meaning in your previous contact with patients or their relatives?<br><br>*How do you deal with it?*		**How do you feel about it?**<br>**What does it do to you?**

Who or what helps you to deal with finding meaning in your own life?<br><br>- *In what form and where (on what topics/issues/area) would you like support?*<br><br>- *What services can you imagine your university offering?*<br>○ *Do they already exist in your eyes? Do you perceive them? What prevents you from doing so?*	**Supporting factors for finding meaning**<br><br>**Personal vs. professional?**<br><br>**Desire for a support offer?**<br>**- Timing of the offer?**<br>**- Scope?**<br>**- Contents?**<br>**- FOCI?**<br><br>**-> Role of the university?**<br><br>**Awareness? Where do participants see this topic already addressed? Obstacles**	**Counter question:**<br>**What is preventing you from engaging in the debate?**<br><br>**So on which topics, questions or areas (own meaning, patients' crises of meaning, meaninglessness in the profession of being a doctor or physician)?**<br><br>**When would you like such a discussion to take place?**<br>**What do you think such support should look like?**
Is there anything else that came up during this interview that you couldn't get rid of?		

- ➔ What role and to what extent do crises of meaning and questions of meaning play in patient contact with students?
- ➔ To what extent and in what form have students of human medicine dealt with questions of finding meaning in the course of their lives before starting their studies and in the course of their training?
- ➔ What changes in questions of finding meaning and a sense of purpose are described by students of human medicine in the course of their medical studies?
- ➔ What is the role of university education in dealing with and supporting the search for meaning?

Status: 30.07.2022
